# The Research on Actuation Performance of MEMS Safety-and-Arming Device with Interlock Mechanism

**DOI:** 10.3390/mi10020076

**Published:** 2019-01-22

**Authors:** Tengjiang Hu, Wei Ren, Yulong Zhao, Yan Xue

**Affiliations:** 1 State Key Laboratory for Manufacturing System Engineering, Xi’an Jiaotong University, Xi’an 710049, China; htj047@xjtu.edu.cn (T.H.); rw0192@163.com (W.R.); 2 Science and Technology on Applied Physical Chemistry Laboratory, Shaanxi Applied Physical Chemistry Research Institute, Xi’an 710061, China; xueyan213@163.com

**Keywords:** MEMS S&A device, electro-thermal actuator, interlock mechanism

## Abstract

Micro-electromechanical systems (MEMS) safety-and-arming (S&A) device shows great potential in munition miniaturization, and it can be seen as the symbol of the fourth generation of weapons systems. In this paper, the design, fabrication, and actuation performance of a silicon based S&A device is presented. It is a multilayer stacked device, which is composed of the cover plate, the actuation chip, and the barrel plate. The electro-thermal principle is investigated in MEMS scale. With 11 V driving voltages, the structure of V-shape actuator, pawl, and slider can generate 100 μm and 45 μm displacement, and realize pulling, disengaging, and reengaging to change the device from the safety position into armed position smoothly (550 μm displacement). The rack and interlock mechanism formed by the pawl and slider gives the device the features of linear output displacement, low power consumption, input signal recognition, and sustained displacement. The 20,000 g setback acceleration is applied, and no structure damage can be found after the impact, which indicates the good anti-load ability of the MEMS S&A device. In order to solve the contradiction between the functional structure and the fabrication process, different structures are designed separately on different wafers. Both silicon and SOI wafers are used in the fabrication process, and the S&A device has been minimized into 8.5 mm × 8.5 mm × 0.8 mm successfully.

## 1. Introduction

Micro-pyrotechnics refers to a new generation of pyrotechnics, which is integrated with energetic material and a micro ignitor. Its miniaturized structure and high output energy density make it to be an important support to the development of weapon miniaturization. As the most sensitive part in the munition system, its security needs to be guaranteed. The traditional Safety-and-Arming (S&A) device can hardly meet the demand of system miniaturization, while micro-electromechanical systems (MEMS) technology shows a tremendous potential and has been explored in this field recently [1,2].

Three main methods can be found to achieve the fabrication of a MEMS S&A device. The first way is the (Lithographie, Galvanoformung and Abformung) (LIGA) method, which is based on metal substrates [3,4]. In this method, a MEMS metal spring and slide are needed. These structures are set perpendicular to each other to form an interlock mechanism. It can function well when driven by proper set back and rotation acceleration. Since most of the metals are elastic materials, they have good explosion-proof characteristics. However, the metal structures fabricated by LIGA are quite expensive and low precision [3]. Although some researchers used electroplate to replace LIGA [5,6], the large structure size and low fabrication precision still limits the development of device miniaturization. The second way is the pyrotechnics method [7,8]. Based on the micro-pyrotechnical principle, high contact quality between heater and energetic material is required. The most difficult point in this method is the compatibility of different fabrication processes (MEMS structure and the energetic material). The currently feasible method is to fill the energetic material in the last step manually [8], which makes it easy to break the structure that has already been done and bring the unsafe factor. The third way is the silicon method. Due to the particularity of the application field, the ultimate output displacement will be larger than 300 μm. In terms of different actuation methods, comb drives and thermal actuators are commonly used. Electrostatic motors respond quickly [9,10]. However, the low output force and high applied voltage restrict their application in S&A devices. Actuators driven by electro-thermal principle have the characteristics of large output force and low applied voltages [11,12]. Steven S. Mink [13] and Robert A. Lake [14] can actuate the silicon S&A device successfully, while the chip size can be minimized into millimeter utilizing surface micromachining technology. However, confined by the fabrication limitation, the thickness of the S&A device was no more than 3.5 μm, which made the whole system too fragile. In summary, the LIGA method is costly and low precision; the pyrotechnics method is unsafe and may cause fabrication incompatibility between the mechanical structure and the energetic material; the silicon method can satisfy the requirement of miniaturization, while the thin thickness limits its development. 

Herein, based on our previous research [15], we will present a novel silicon based MEMS S&A device. Different from the silicon method mentioned above, bulk micromachining is introduced in the fabrication process, which can enlarge the device thickness into 50 μm. The device is composed of the cover plate, the actuation chip, and the barrel plate. These basic components will be bonded together to compose the ultimate MEMS S&A device. The electro-thermal principle is investigated in the device. With specific driven voltages, the MEMS S&A device can realize 550 μm output displacement in 700 ms, while the chip total size is 8.5 mm × 8.5 mm × 0.8 mm.

## 2. Modeling

**Figure 1:**
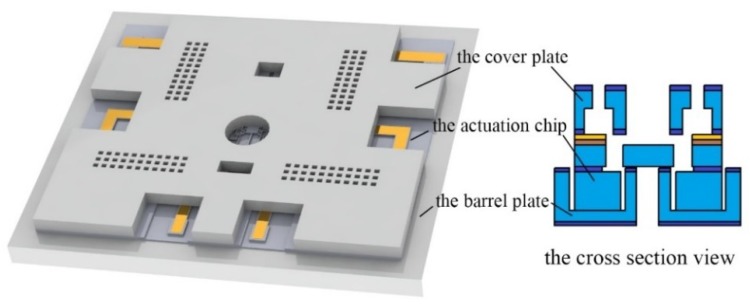
The structure of MEMS safety-and-arming (S&A) device.

The MEMS S&A device should have the basic functions of signal recognition, proper driven movement, and detonation energy transmission. In order to realize these characteristics in the limited chip area, the modularize method is introduced. The S&A device is divided into three parts: the cover plate, the actuation chip, and the barrel plate, shown in Figure 1.

### 2.1. The Cover Plate

**Figure 2:**
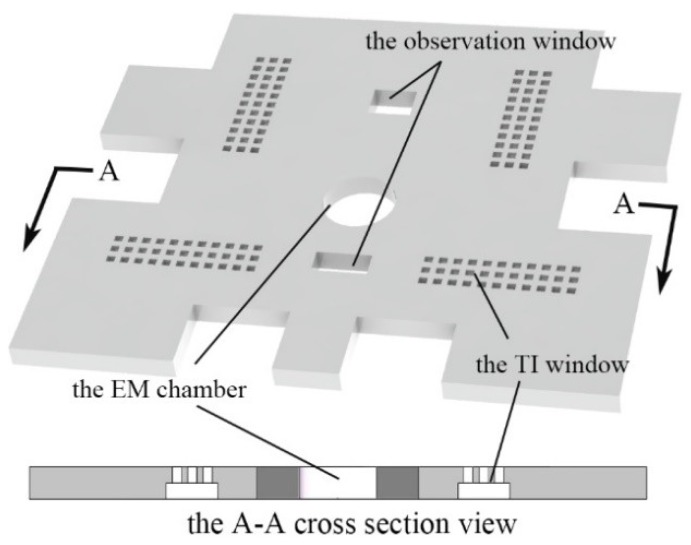
The structure of the cover plate.

The cover plate worked as the encapsulation to protect the MEMS S&A device from the hostile environment. Besides that, the basic requirements of the cover plate are: (1) Constrain the out-off-plane movement of the S&A device. (2) Fix the energetic material. The Energetic Material (EM) chamber is designed in the center of the cover plate to guarantee the integrity of the detonation train. (3) Motion observation. Every step movement of the actuation chip can be monitored through the two observation windows. (4) Thermal Isolation (TI) windows are introduced. Since the S&A device is driven by electro-thermal principles, the structure of TI windows can enhance the thermal efficiency drastically. With all the requirements, the structure of the cover plate is shown in Figure 2.

### 2.2. The Actuation Chip

The actuation chip is the core component of the MEMS S&A device. Considering the workability of the fabrication process, the paper examines the electro-thermal principle. There are three modes of heat transfer: conduction, convection, and radiation, however, in MEMS scale, the convection and radiation can be neglected [16,17]. The heat diffusion analysis is simplified into a one-dimensional problem, and the steady temperature distribution of the V-shape beam is expressed in the following equations:

(1) $$k_{s}\frac{d^{2}T\left(x\right)}{dx^{2}}+J^{2}\rho =\frac{S}{h}\frac{T\left(x\right)-T_{r}}{R_{T}}$$

with the thermal boundary conditions:

(2) $$T\left(0\right)=T\left(L\right)=T_{r}$$

Here, *k_s_* is the thermal conductivity of silicon. *J* stands for the electrical current density. *V* represents the applied voltage. *θ* is the angle of the V-shape beam and *ρ* is the electrical resistivity of silicon. *L* refers to the length of the V-shape beam. *R_T_* = *t_V_/k_V_* is the thermal resistance between the bottom of the structure and the surface of the substrate, which can affect the heat dissipation of the device drastically. The air thermal conductivity *k_V_* is the material property, which means the parameter *R_T_* is mainly based on the structure size *t_V_*. Without the TI window, *t_V_* is equal to the thickness of the buried layer (3 μm), while with the TI window, *t_V_* equals the thickness of the substrate (403 μm), and the parameter *R_T_* can be enlarged almost 134 times. Just like the concept of resistance, the larger *R_T_* is, the harder thermal conduction will be, and the heat loss between the heater and the substrate can be inhibited effectively. As the shape factor, *S* is used to evaluate the effect of the structure size on heat transfer [18,19]. *T_r_* is the reference temperature, and usually it equals the room temperature. 

**Figure 3:**
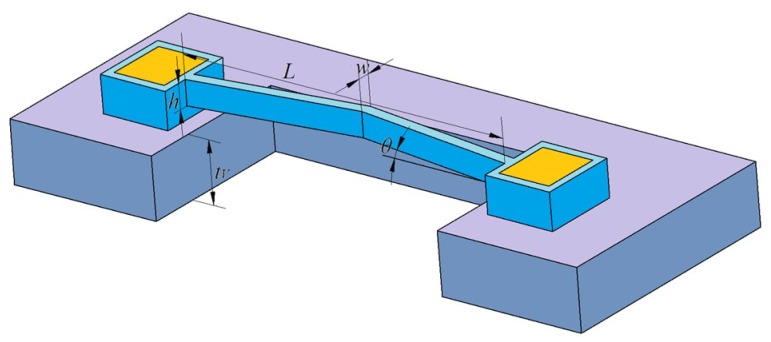
The structure of the V-shape electro-thermal actuator.

The thermal expansion generated by the V-shape electro-thermal actuator, shown in Figure 3, can be expressed as Equation (3):

(3) $$d=\frac{L}{2}\left(\sqrt{\frac{{\left(1+\alpha \left(T-T_{r}\right)\right)}^{2}}{\cos^{2}\theta }-1}-\tan\theta \right)$$

here, α is the thermal expansion coefficient of silicon. *T* is the average temperature of the beam.

Considering the complexity of the MEMS S&A device, the optimization of the whole device will be a huge work. Thus, we just optimized the key component (the actuator). The optimization module in ANSYS is used in this part. The objective function is the minimum occupied area of the actuator. The maximum temperature (<1200 K), the maximum stress (<1 GPa), and the minimum displacement (>200 μm) are the design constraints. According to the requirements of our collaborator, the driven voltage (11 V) and the size of the micro lever are fixed, and the optimization variables are *L* and *w* of the actuator. The optimization results are listed in Table 1.

**Figure 4:**
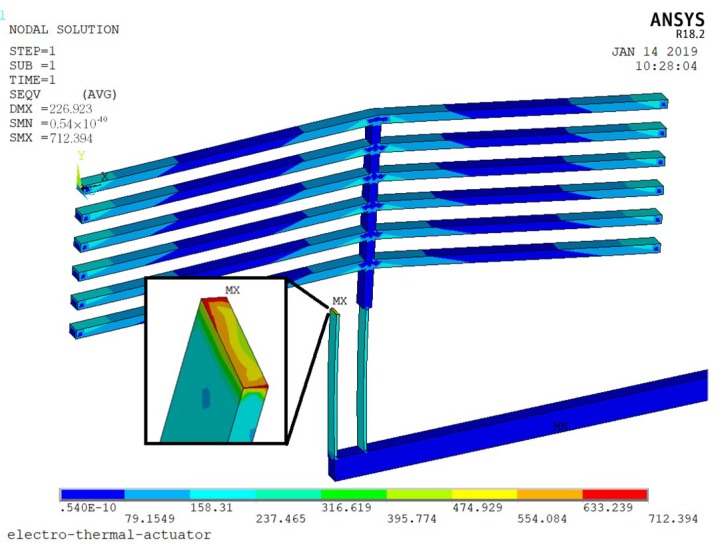
The maximum stress of the actuator.

The data in Set8 are the optimized geometry parameters of the actuator. The result shows that the maximum displacement is about 223.7 μm when 11 V driven voltage is applied. The maximum temperature occurs in the middle of the V-shape beam, which is 957.17 K. The maximum stress is 636.84 MPa, which occurred in the flexible beam, shown in Figure 4.

Since the thermal expansion of silicon is quite small, the tiny deformation of the V-shape beam can hardly be used in the S&A field. Thus, displacement amplifier mechanism should be investigated in the designing section. The micro lever is an available solution in MEMS scale, however, constrained by the structure stiffness, the amplifier coefficient of micro lever is limited. In order to achieve the requirement of 500 μm output displacement, rack mechanism is introduced. Just like the gear mechanism, the rack mechanism is comprised of the pawl and slider. By accumulating the small deformation of every step, the large output displacement can be realized. 

**Figure 5:**
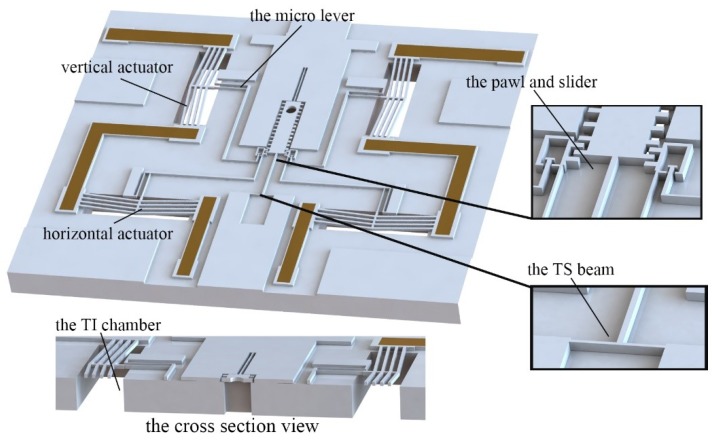
The structure of the actuation chip.

The basic structure of the actuation chip is shown in Figure 5. Two pawl actuators are designed as axisymmetric in the structure, which can actuate the slider alternatively. The pawl actuator is composed of a horizontal actuator and vertical actuator. The horizontal actuator is designed to pull the slider; the vertical actuator is designed to realize the motion of disengagement and reengagement between the pawl and slider. The teeth on the pawl and slider will lock to each other and the interlock function can be achieved. In order to enhance the output performance of the electro-thermal actuator, TI chambers are also designed in the substrate of the actuation chip. Although the slider is the free moving part in the device, it is fixed to the structure by the Temporary Support (TS) beams. When covered by the cover plate, the TS beams will be broken up by the probe, and then the slider is free to move.

**Figure 6:**
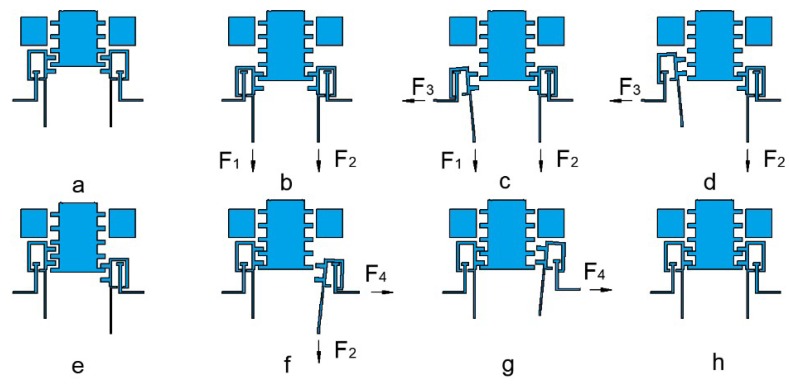
The operation process of the actuation chip. (**a**) Initial state. (**b**) Actuator No.2 and No.3 pull the slider to move one sub-step. (**c**) Actuator No.1 disengages the left pawl and slider. (**d**) Teeth on the left pawl align with the gap on the slider. (**e**) The left pawl reengages with the slider. (**f**) Actuator No.4 disengages the right pawl and slider. (**g**) Teeth on the right pawl align with the gap on the slider. (**h**) The right pawl reengages with the slider and accomplishes one step movement.

All of the actuators are marked from No.1 to No.4 to give a clear instruction of the operation process, shown in Figure 6a.

First, 11 V voltage is applied on actuator No.2 and No.3 to pull the slider to move 1 sub-step length (about 100 μm), shown in Figure 6b.

Second, voltage is applied on actuator No.1 to disengage the left pawl from the slider. The disengagement displacement is about 40 μm, shown in Figure 6c.

Third, the applied voltage on actuator No.2 will be removed which makes the teeth on the left pawl align with the gap on the slider, shown in Figure 6d.

Fourth, the applied voltage on actuator No.1 will be removed which makes it return to its original position, shown in Figure 6e.

Fifth, voltage is applied on actuator No.4 to make the right pawl disengage from the slider, shown in Figure 6f.

Sixth, the applied voltage on actuator No.3 will be removed which makes the teeth on the right pawl align with the gap on the slider, shown in Figure 6g.

Seventh, the applied voltage on actuator No.4 will be removed to make the teeth on the right pawl reengage with the slider, shown in Figure 6h. Now, the four actuators will get back to the initial place and get ready for the next step. 

**Figure 7:**
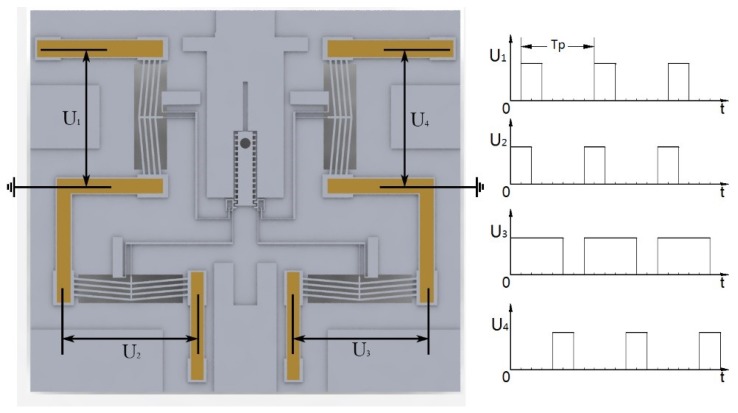
The driven signal of the actuation chip.

From the operation process, it is obvious to see that there will always be at least one of the pawls engaged with the slider. This special design forms the interlock mechanism of the device, which means the slider can only be actuated by the specific signal, otherwise the slider will stay in the previous position. The specific driven signal is shown in Figure 7. 

### 2.3. The Barrel Plate

The barrel plate is another necessary part of MEMS S&A device. It is mainly used to solve the contradiction between the functional structure and the fabrication process. In order to make sure the slider can be released thoroughly, the silicon substrate that is underneath the slider needs to be removed. However, the huge rectangle chamber left on the silicon substrate will sabotage the integrity of the explosion train. Thus, the silicon barrel plate is introduced in the MEMS S&A device.

**Figure 8:**
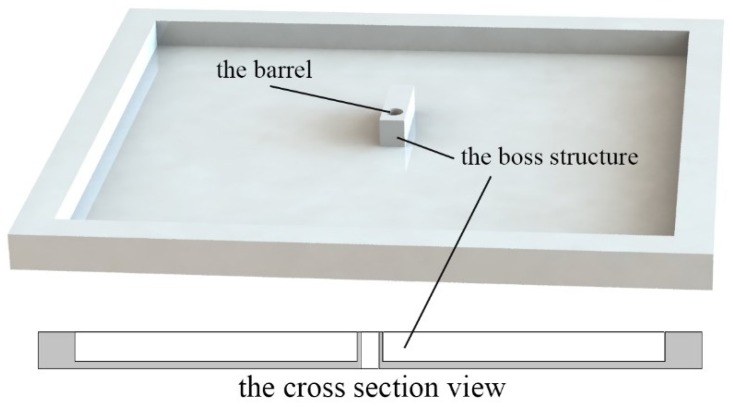
The structure of the barrel plate.

The 400 μm (equals to the substrate thickness of the actuation chip) boss structure is placed in the center of the barrel plate, and the *ɸ* 500 μm barrel is designed based on the boss structure. In order to simplify the assembly process, the frame structure is designed around the barrel plate, shown in Figure 8.

## 3. Fabrication Process

**Figure 9:**
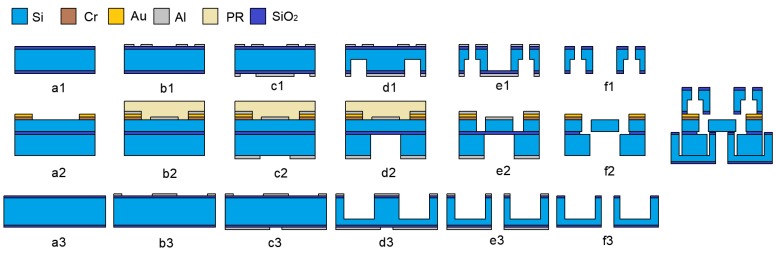
The fabrication processes. (**a1**) Insulation layer. (**b1**) Front mask layer. (**c1**) Backside mask layer. (**d1**) Backside etching. (**e1**) Front etching. (**f1**) Cleaning. (**a2**) Bond pad layer. (**b2**) Structure mask layer. (**c2**) Backside mask layer. (**d2**) Handle layer etching. (**e2**) Structure layer etching. (**f2**) Releasing. (**a3**) Insulation layer. (**b3**) Front mask layer. (**c3**) Backside mask layer. (**d3**) Backside etching. (**e3**) Front etching. (**f3**) Cleaning. The cover plate, the actuation chip, and the barrel plate were bonded together by the high temperature adhesive.

The MEMS S&A device is fabricated on silicon wafer and silicon-on-insulator (SOI) separately. All of the wafers are double-side polished and detailed parameters are shown in Table 2, and the main fabrication steps are illustrated in Figure 9.

The fabrication process of the cover plate: (a1) Insulation layer. 300 nm SiO_2_ layer was deposited on both sides of the silicon wafer; (b1) Front mask layer. 200 nm layer of Al was patterned by lift-off; (c1) Backside mask layer. Another layer of 200 nm Al was patterned on the backside of the wafer; (d1) Backside etching. The SiO_2_ layer formed in step a1 was removed by buffered oxide etchant (BOE), and the exposed Si was etched by inductively coupled plasma (ICP) (100 μm in depth). The cavity structure formed in this step was used as the EI chamber, which can increase the thermal resistance and enhance the thermal efficiency drastically; (e1) Front etching. The wafer was etched from the front side to the backside by ICP, and the observation windows and the EM chamber are formed; (f1) Cleaning. The Al layer was removed.

The fabrication process of the actuation chip: (a2) Bond pad layer. Bond pad (Cr/Au 50 nm/300 nm) was placed on device layer using lift-off; (b2) Structure mask layer. 400 nm layer of Al was sputtered and patterned as the structure mask, and then protected by the photo resist (PR); (c2) Backside mask layer. 400 nm layer of Al was deposited and patterned on the handle layer; (d2) Handle layer etching. The silicon on the handle layer was etched to the buried layer (400 μm in depth); (e2) Structure layer etching. The structure layer was etched to the buried layer by ICP (50 μm in depth); (f2) Releasing. The whole wafer was diced into separated chips and released by hydrofluoric acid (HF). Considering the slider may probably flow away in HF etchant, TS beams are designed in the structure

The fabrication process of the barrel plate was similar to that of the cover plate, which included an insulation layer, front mask layer, backside mask layer, backside etching, front etching, and cleaning. Then, the cover plate, the actuation chip, and the barrel plate were bonded together by the high temperature adhesive. 

**Figure 10:**
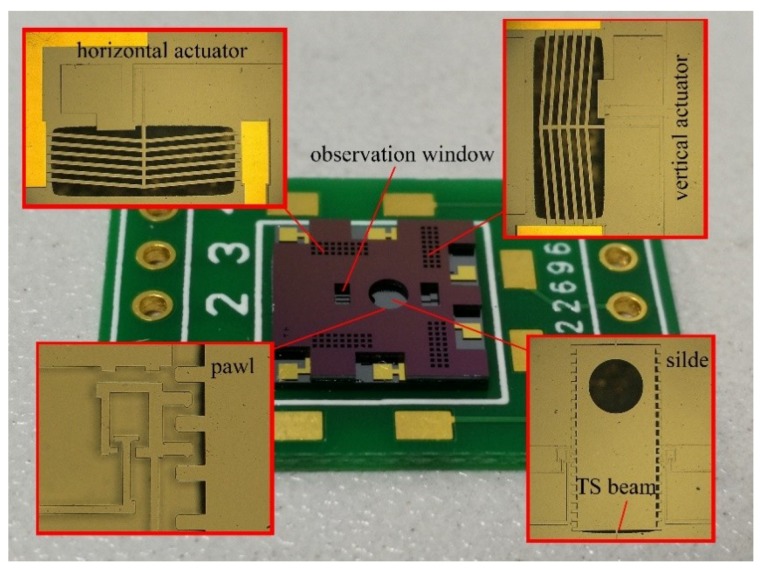
Detail structure of the S&A device.

More detail of the structure can be seen in Figure 10. Confined by the cover plate, the movable slider and the suspended structure in the actuation chip are well protected. The TI chamber was fabricated beneath the V-shape beam to guarantee the proper thermal resistance of the device. The slider is designed as a free-moving component, and it may probably drop out of the actuation chip during the releasing process. In order to avoid that, the slider is fixed to the substrate by the TS beams temporarily. The TS beams are designed as slender beams and can easily form the stress concentration area at the structural joints. Once protected by the cover plate, the TS beams can be broken by the probe (through the observation window designed on the cover plate). Without constraint by the TS beams, the slider can be fully released.

## 4. Tests of Actuation Performance

### 4.1. The Actuation Froce

**Figure 11:**
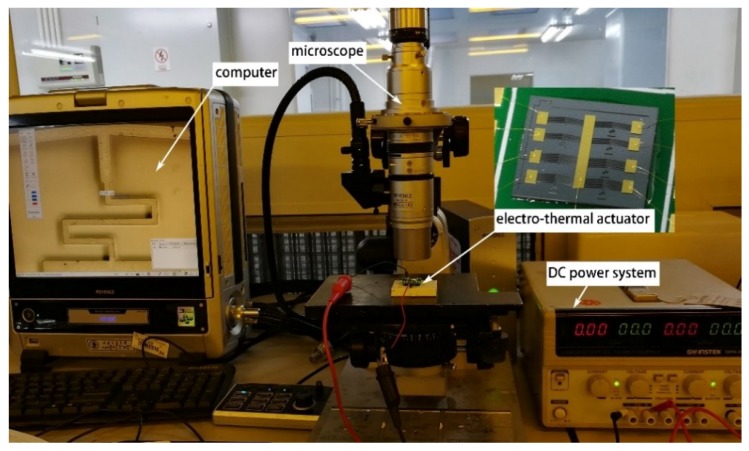
The measurement stage of the actuation force.

Compared to the displacement measurement of the electro-thermal actuator, it is hard to obtain the data of the output force. Herein, a micro spring is introduced and the measurement stage is shown in Figure 11. The stage was composed of a direct current (DC) power system (GPC-6030D), electro-thermal actuator, microscope (KEYENCE-VHX-2000, Keyence Corporation, Osaka, Japan), and computer. When the driving signal was applied, the actuator will pull the micro spring to deform. By measuring the spring’s displacement, the actuation force can be calculated.

**Figure 12:**
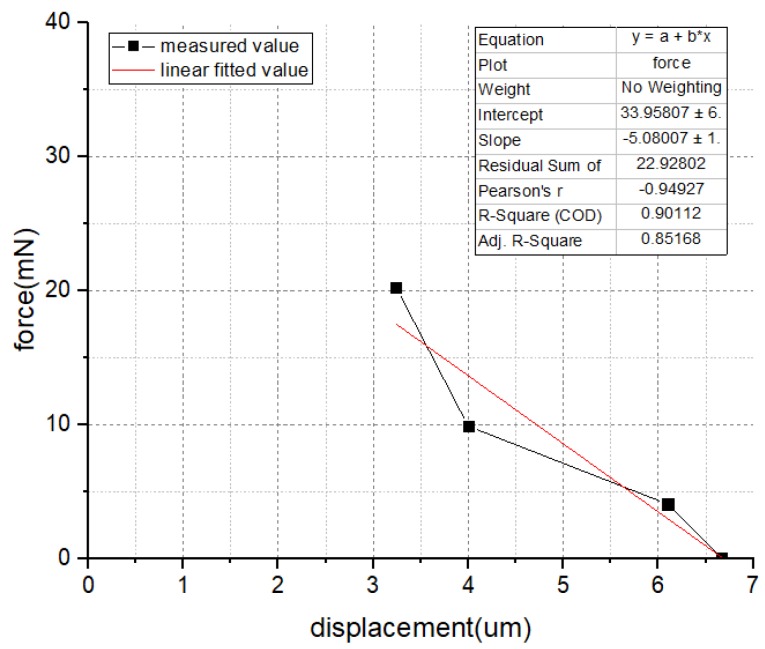
The measurement result of the actuation force (single V-shape actuator).

In order to obtain the measurement data more precisely, four springs with different coefficients of stiffness were introduced, which were 0 N/m, 665.8 N/m, 2466 N/m, and 6249.5 N/m, respectively. The measurement results were shown in Figure 12. The dark spots were the measured value, and the red line was the linear fitted value (the spring was a linear system). The maximum actuation force occurred when the displacement was suppressed into zero, which means the value is equal to the Y-intercept of the fitted line. Take the single V-shape actuator (16 V driven voltage) as an example, the measurement result was 33.96 mN.

### 4.2. The Displacement of V-shape Actuator

**Figure 13:**
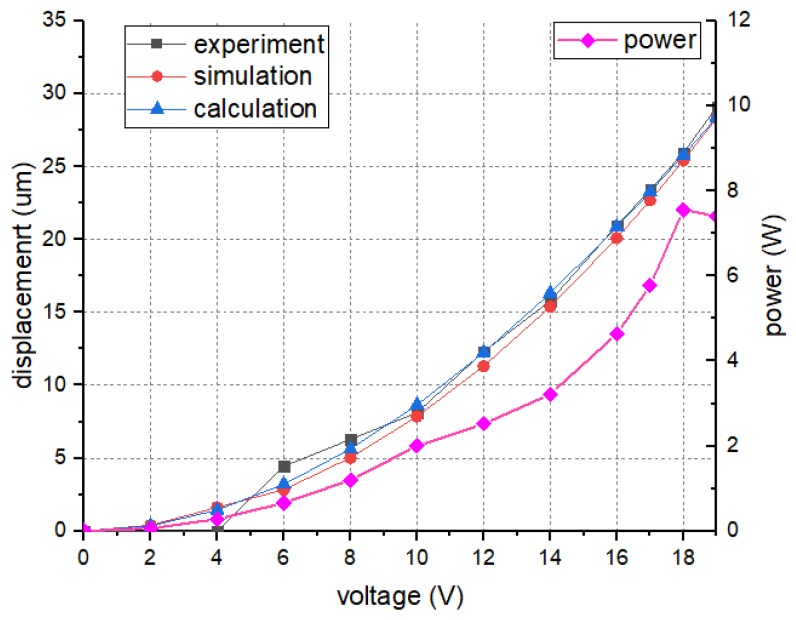
The test results of the electro-thermal actuator.

The performances of the V-shape electro-thermal actuator are investigated. The direct voltage is applied on the device. The results of displacement and power consumption are shown in Figure 13. The test results indicate that higher voltage can lead to larger displacement. Although the working voltage is restricted in 11 V, the actuator can still function under 19 V, and the maximum displacement is 29 μm. The experiment results are in good agreement with the theoretical and simulation results, which are 28.4 μm and 28.3 μm, and the deviations are 2.1% and 2.4%, respectively. The electro-thermal beam will be over-heated and burned down under 20 V. In order to guarantee the device safety, the applied voltage is restricted in 11 V, and the power consumption is 2.26 W.

### 4.3. The Response Time

**Figure 14:**
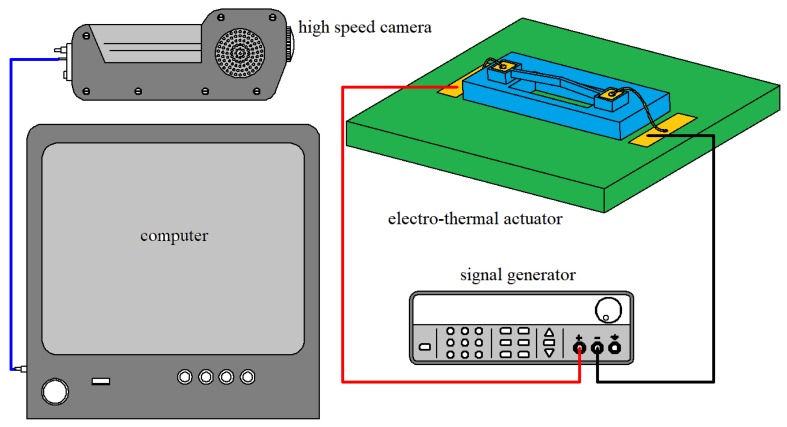
The measurement stage of the response time.

The measurement stage of the response time was composed of a high speed camera (PHANTOM M320S, Phantom Inc., Bublin, OH, USA), resistor, electro-thermal actuator, computer, and the signal generator (UTG2062B, UNI-T, Dong Guan, China). The high speed camera (1000FPS) and the electro-thermal actuator were triggered by the signal generator simultaneously. By analyzing the image data captured from the camera, the step response time of the electro-thermal actuator can be obtained. A 1.5 s actuation time was set to guarantee the actuator reached its steady state, and the measurement result of the step response time is about 16 ms, shown in Figure 14.

### 4.4. The Moving Tests

As mentioned above, the S&A device can be actuated only under the specific signal, thus, the control circuit needs to be designed. Considering the strict phase relationship between driving signals, a 555 multi-vibrator is needed. Similar to the conductor of the symphony orchestra, 555 can provide a stable square wave signal, which can be seen as the reference. By counting the peak of the signal, the logical relationship between the control signal can be obtained accurately and quickly, shown in Table 3.

With the help of Table 3, the logic expression of the 4 control signals are:

(4) $$U_{1}=B\bar{C}$$

(5) $$U_{2}=A\bar{B}\bar{C}+\bar{A}B\bar{C}=\left(A⊕B\right)\bar{C}$$

(6) $$U_{3}=A\bar{B}+\bar{B}C+B\bar{C}=A\bar{B}+\left(B⊕C\right)$$

(7) $$U_{4}=\bar{A}BC+A\bar{B}C=\left(A⊕B\right)C$$

**Figure 15:**
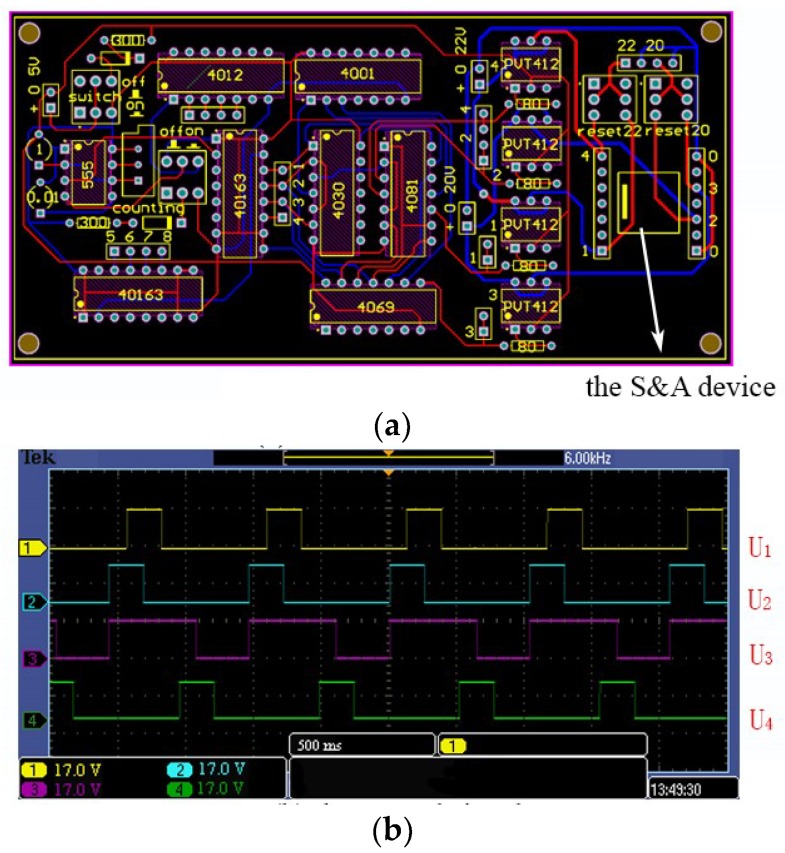
The control circuit and the control signal. (**a**) The control circuit. (**b**) The control signal.

The control circuit is shown in Figure 15. The S&A device is encapsulated in dual in-line package (DIP) form, and by plugging into the signal hole, the whole device can be controlled automatically. The actuation time depends on the time period and the step. The number of the step is fixed into 5, while the time period is affected by the resistance (R) and the capacitance of the control circuit (C). Since *T*_period_ *=* 0.*7 R* × *C*, the change of R and C can change the *T*_period_ drastically. However, confined by the electro-thermal principle, the time period should not be shorter than the response time (16 ms), otherwise, the S&A device can’t be actuated successfully.

**Figure 16:**
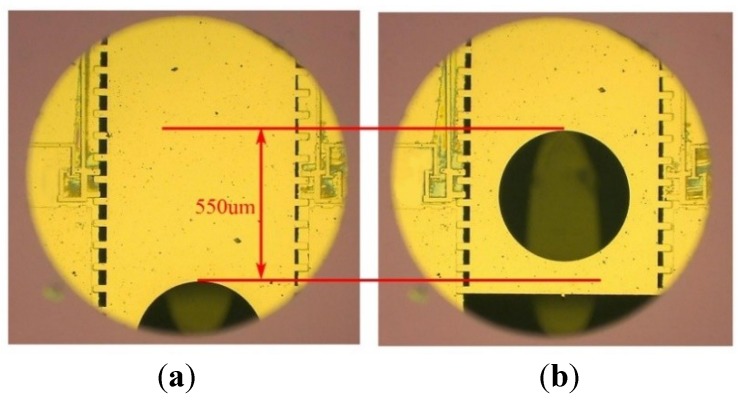
The movement results of the S&A device. (**a**) The safety position. (**b**) The armed position.

The actuation performances of the S&A device are investigated in this part. When 11 V driving signals were applied in the specific sequence, the whole device could function well and the slider could be moved smoothly. Every step movement is shown in Figure 16, and its performance matched the prediction. The pulling displacement is measured as 100 μm, and the disengagement displacement is 45 μm. In Figure 15b, two pawl actuators have returned to their initial place, while the slider has accomplished 550 μm movement, which means the S&A device has already turned the safety position into the armed position. The whole function time of the device is depended on *T*_period_ of the signal, and can be modified under different working situations (the minimum T_period_ = step response time × number of sub step × number of step = 20 × 7 × 5 = 700 ms). Due to the interlock mechanism, the slider will stay in its position without extra energy. 

**Figure 17:**
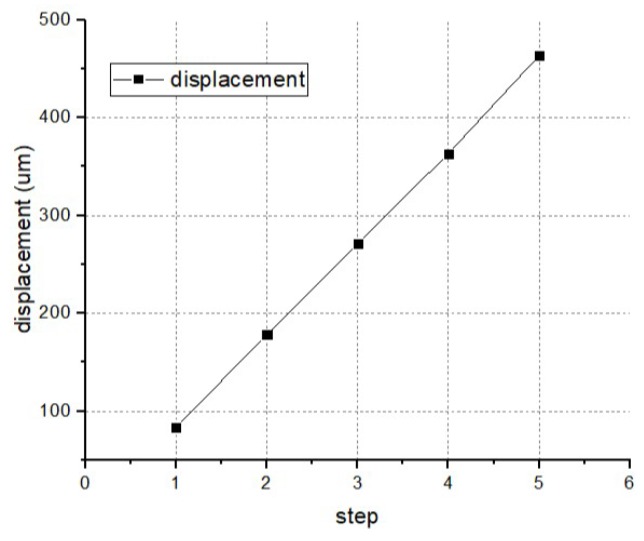
The output displacement versus the moving step.

The electro-thermal actuator is a typical nonlinear system, and the output displacement can increase drastically with the input voltage, which is inconvenient for the device motion control monitoring. The rack mechanism, which is composed of the pawl and slider, can solve this problem. Since the teeth spacing on the slider is fixed, the movement of every step can be determined, and the finial output displacement is linear with the moving steps, shown in Figure 17.

### 4.5. The Anti-Load Tests

**Figure 18:**
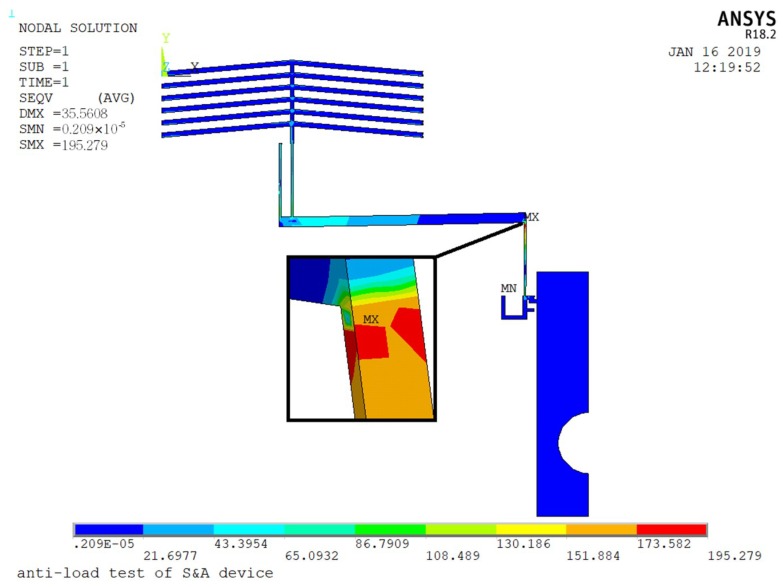
The maximum stress of the MEMS S&A device (20,000 g setback acceleration).

During the launching period, the whole weapon system will withstand certain environmental forces. In order to ensure the system can function normally after the impact, the S&A device needs to have the ability of anti-load. Considering the size of the MEMS structure is quite small, the effect of the environmental acceleration is limit. The largest movable part in the MEMS S&A device is the slider (0.85 mm × 2 mm × 0.05 mm), which weights 0.2 μg. Under the 20,000 g setback acceleration (*y* direction), the inertia force is calculated as 39.2 mN. Since the structure of the MEMS S&A device is symmetric (half model was applied here), the inertia force will be applied on the electro-thermal actuator evenly (19.6 mN), and the maximum stress caused by this is 195.27 MPa, which is much lower than the breaking stress of silicon (7 GPa), shown in Figure 18.

**Figure 19:**
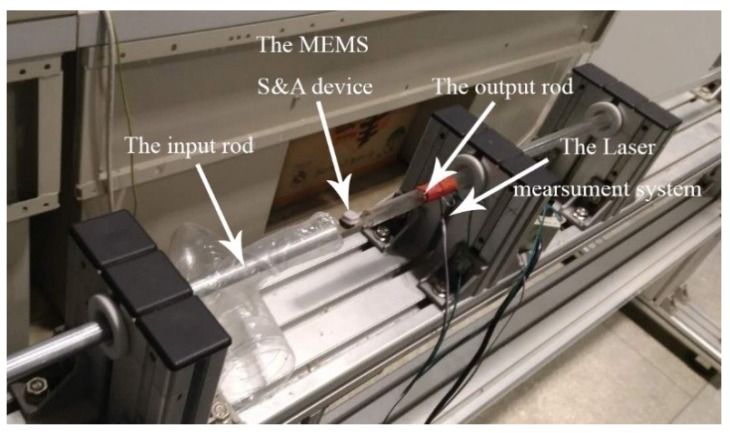
The Hopkinson pressure rod system.

The experiment stage of the anti-load test was composed of a high speed camera (PHANTOM M320S), the MEMS S&A device, computer, and the Hopkinson pressure rod (SHPB-ALT-1000, ARCHIMEDES, Berlin, Germany). The Hopkinson pressure rod was mainly composed of the gas storage system, the impact rod system, the laser speed measurement system, the output rod, and the data acquisition system, shown in Figure 19. In the experiment, the MEMS S&A device was connected to the distal end of the output rod, the gas storage system pushed the impact rod and collided with the proximal end of the output rod. Meanwhile the laser speed measurement system and the data acquisition system recorded and calculated the acceleration during the impact.

**Figure 20:**
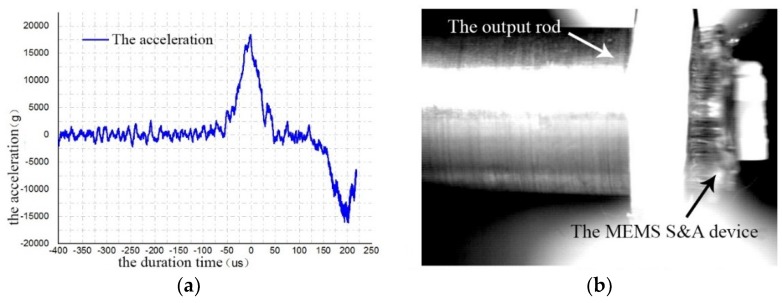
The anti-load test result of the S&A device. (**a**) The setback acceleration; (**b**) the Figure captured by the high speed camera.

The anti-load test result of the S&A device was shown in Figure 20. The peak value of the acceleration was 20,000 g, and the duration was 100 μs. After the impact, the MEMS S&A device had been detached from the output rod. However, there was no structure damage inside, and the whole S&A device can still function well. 

## 5. Discussions and Conclusions

The design, fabrication, and actuation performance of a silicon based S&A device is presented in this paper. It is a multilayer stacked device, which is composed of the cover plate, the actuation chip, and the barrel plate. The electro-thermal principle is investigated in MEMS scale. With 11 V driving voltages, the structure of V-shape actuator, pawl, and slider can generate 100 μm and 45 μm displacement, and realize pulling, disengaging, and reengaging to smoothly change the slider from the safety position into armed position (550 μm displacement). The rack and interlock mechanism formed by the pawl and slider give the device the features of linear output displacement, low power consumption, input signal recognition, and displacement sustaining. The 20,000 g setback acceleration is applied, and no structure damage can be found after the impact, which indicates the good anti-load ability of the MEMS S&A device.

The S&A device introduced in this paper has been minimized into 8.5 mm × 8.5 mm × 0.8 mm successfully. As one of the core components of the munition system, the micro S&A device should be integrated with a micro detonator and micro energetic material. Currently, the research on both fields have also received much attention, and the fabrication processes are developing in the direction of compatibility with MEMS process. With a micro detonator and micro energetic material, the S&A device could be more practical, which is worthwhile to investigate in future work.

## Figures and Tables

**Table 1:** The optimized results.

Parameters	Set1 Infeasible	Set2 Feasible	Set3 Feasible	Set4 Feasible	Set5 Feasible	Set6 Feasible	Set7 Feasible	*Set8* Feasible
Smax (MPa)	617.05	638.20	645.39	644.27	638.55	638.21	620.59	636.84
Dmax (μm)	183.85	257.85	235.52	243.16	227.62	224.62	223.90	223.70
*L* (μm)	1600	2338.8	2155.2	2215	2044.8	2011.6	2003.8	2001.6
*w* (μm)	20	34.572	36.924	36.179	31.403	30.341	30.103	30.037
Volume (μm^3^)	0.1887 × 10^8^	0.33468 × 10^8^	0.33054 × 10^8^	0.33232 × 10^8^	0.28478 × 10^8^	0.27531 × 10^8^	0.27318 × 10^8^	0.27259 × 10^8^

**Table 2:** Parameters of silicon wafer and silicon-on-insulator (SOI).

The Cover Plate (Silicon Wafer)		The Actuation Chip (SOI Wafer)			The Barrel Plate (Silicon Wafer)	
Parameters	Specification	Parameters		Specification	Parameters	Specification
Diameter (mm)	100	Diameter (mm)		100	Diameter (mm)	100
Orientation	(100)	Orientation		(100)	Orientation	(100)
Resistivity (Ω∙cm)	1–20	Resistivity (Ω∙cm)	Device layer	0.01–0.02	Resistivity (Ω∙cm)	1–20
			Handle layer	1–20		
Thickness (μm)	300	Thickness (μm)	Device layer	50	Thickness (μm)	500
			Buried oxide layer	3		
			Handle layer	400		

**Table 3:** The control signal.

Step (Decimalism)	Step (Binary)			U_1_	U_2_	U_3_	U_4_
N	C	B	A				
0	0	0	0	0	0	0	0
1	0	0	1	0	1	1	0
2	0	1	0	1	1	1	0
3	0	1	1	1	0	1	0
4	1	0	0	0	0	1	0
5	1	0	1	0	0	1	1
6	1	1	0	0	0	0	1
7	1	1	1	0	0	0	0
